# FunFOLD: an improved automated method for the prediction of ligand binding residues using 3D models of proteins

**DOI:** 10.1186/1471-2105-12-160

**Published:** 2011-05-16

**Authors:** Daniel B Roche, Stuart J Tetchner, Liam J McGuffin

**Affiliations:** 1 School of Biological Sciences, University of Reading, Whiteknights, Reading RG6 6AS, UK

## Abstract

**Background:**

The accurate prediction of ligand binding residues from amino acid sequences is important for the automated functional annotation of novel proteins. In the previous two CASP experiments, the most successful methods in the function prediction category were those which used structural superpositions of 3D models and related templates with bound ligands in order to identify putative contacting residues. However, whilst most of this prediction process can be automated, visual inspection and manual adjustments of parameters, such as the distance thresholds used for each target, have often been required to prevent over prediction. Here we describe a novel method FunFOLD, which uses an automatic approach for cluster identification and residue selection. The software provided can easily be integrated into existing fold recognition servers, requiring only a 3D model and list of templates as inputs. A simple web interface is also provided allowing access to non-expert users. The method has been benchmarked against the top servers and manual prediction groups tested at both CASP8 and CASP9.

**Results:**

The FunFOLD method shows a significant improvement over the best available servers and is shown to be competitive with the top manual prediction groups that were tested at CASP8. The FunFOLD method is also competitive with both the top server and manual methods tested at CASP9. When tested using common subsets of targets, the predictions from FunFOLD are shown to achieve a significantly higher mean Matthews Correlation Coefficient (MCC) scores and Binding-site Distance Test (BDT) scores than all server methods that were tested at CASP8. Testing on the CASP9 set showed no statistically significant separation in performance between FunFOLD and the other top server groups tested.

**Conclusions:**

The FunFOLD software is freely available as both a standalone package and a prediction server, providing competitive ligand binding site residue predictions for expert and non-expert users alike. The software provides a new fully automated approach for structure based function prediction using 3D models of proteins.

## Background

Because of a protein's essential cellular role, it is important to fully understand its structure and interactions. Predicting the location of the binding site and the ligand binding residues, is a necessary step towards elucidating how a protein functions [1]. The determination of the ligand binding site residues in a protein is also important, because substrate specificity of an enzyme is determined by the fine details of the binding site residues, such as side chain orientation and physiochemical properties [2].

Several different protein ligand binding site prediction methods have been developed, which mostly fall into two major categories: sequence-based methods and structure-based methods [3]. The sequence-based methods rely on identifying conserved residues that may be structurally or functionally important and include methods such as firestar (CASP9 group FN315) [4], WSsas [5], FRcons [6], ConFunc (CASP8 - FN437) [7], ConSurf [8], FPSDP (CASP8 - FN242) [9] and INTERPID [10]. The structure-based methods can be further subdivided into the geometric methods utilised by FINDSITE [11] and SiteHunter (CASP8 - FN163) [12]; energetic methods utilised by SITEHOUND [13] and Q-SiteFinder [14] and miscellaneous methods that utilise information from; homology modelling, such as 3D LigandSite [15] (CASP9- FN017, FN415, FN057, FN072), Mariner1 (CASP8 - FN450) [16] and MetSite [17]; surface accessibility used by the LIGSITE^csc ^[18] method and physiochemical properties utilised by SCREEN [19].

In the CASP6 experiment, a function prediction category was included for the first time, where groups were required to predict Enzyme Commission numbers (EC) and Gene Ontology (GO) terms [20]. As a result of the difficulty in assessing these terms, the CASP7 assessors made a decision to modify the function prediction category [21]. Consequently in CASP8, the function prediction was included in a different format, with ligand binding site residues assessed, as many CASP targets were found to crystallize with biologically relevant ligands [1].

In CASP8, the top methods in the function prediction category were the methods by the Lee group [3] and the Sternberg group [22]. The Lee group had two main methods: a manual method (FN407) and an extended deadline server method (FN293). The Lee extended deadline server method relied on manual configuration of distance cut-offs for each target and, at the time of writing, a server based on this method is not publicly available. The Sternberg group was also registered as a manual prediction group (FN202), however since CASP8, the group has produced a publicly available server, 3D LigandSite, which is reported to be similar in performance to their manual prediction method from CASP8 [15].

The Lee group's manual and server methods from CASP8 only differed in the top 3D models used for prediction. The methods were both based on 3D superposition of structurally similar proteins that contain ligands. Residues were considered to be in contact with the ligands if the distance between them was less than 0.5Å + the Van der Waals radii, and the cut-offs for including residues in predictions were manually altered depending on the target [3].

The Sternberg group's manual predictions in CASP8, and their 3D LigandSite [15] server, used a very similar method [22] to the Lee group for ligand binding site prediction. The Sternberg group used the 3D-Jury method [23] in order to select from amongst the CASP8 server models followed by structural superposition of models and related templates. In addition, residue conservation was considered using ConFunc [7].

However, for the 3D LigandSite [15] server method, Wass *et al*. used the top 3D models from the Phyre server [24] and MAMMOTH [25] for structural superposition of similar templates with bound ligands. The residue conservation scoring was found to cause significant over prediction of residues [22] and so data from ConFunc is not directly included in the current 3D LigandSite server predictions [15]. To the best of our knowledge the 3D LigandSite server is the best publicly available fully automated method for prediction of ligand binding residues that has been independently benchmarked on the CASP8 data set and it produces comparable results to that of group FN202 [15]. Hence in this study we firstly compare our novel approach, FunFOLD, against all the groups at CASP8, paying particular attention to the comparison with the predictions from CASP8 group FN202.

In CASP9, the top few ranked methods in the function prediction category were by the Zhang group (FN096, FN339) and the firestar group (FN035, FN315). The I-TASSER-FUNCTION and Zhang human methods are again based on model-to-template superposition, to predict the ligand binding site residues. However, at the time of writing no publicly available server has been produced that implements the I-TASSER-FUNCTION method. The firestar (FN315) server, which is publicly available, also utilized sequence conservation from PSI-BLAST [26] profile alignments in order to predict ligand binding site residues [4].

According to the official CASP9 assessment [27], it was difficult to find statistically significant separations between the top 11 or so groups in the function prediction category, which included - Zhang (FN096), I-TASSER-FUNCTION (FN339), firestar (FN315), FAMSSEC (FN113), CNIO-firestar (FN035), Sternberg (FN110), Seok (FN242), Jones-UCL (FN104), Seok-server (FN452), Lee (FN114), McGuffin (FN094) and gws (FN236). Hence we also compare FunFOLD against all groups at CASP9, paying particular attention to the comparison with these top performing groups and their associated servers: I-TASSER-FUNCTION (FN339), firestar (FN315), Seok-server (FN452), gws (FN236) and the 3D LigandSite servers (FN017, FN415, FN057, FN072).

The FunFOLD method is similar in concept to other groups' methods, such as the Lee group and the Sternberg group methods, which use protein structure superposition of distantly related templates to a modelled protein to identify ligand binding sites. However, the FunFOLD algorithm uses a novel automated method for ligand clustering and identification of binding residues. We also investigate the use of ModFOLDclust2 [28] to select different starting models from amongst the pool of alternative server models and gauge the affect on accuracy.

## Implementation

### FunFOLD

The FunFOLD method for predicting ligand binding site residues is based on the concept that, ligand containing templates from the PDB with the same folds (according to TMalign [29]) as the 3D model of the target protein under analysis, may contain similar binding sites. The FunFOLD standalone method takes as its input a 3D model of the protein under analysis and a list of template PDB IDs, which can be obtained from the templates used to build the model. The prototype version of the FunFOLD server method, which was developed during the CASP9 prediction season (listed as the group "IntFOLD-FN" - FN425 (server)), queried the FireDB [30] in order to identify if ligands in each template PDB file were biologically relevant. This method worked well, but it relied on the FireDB database, which has been updated since CASP8. Although it was unlikely that the identified biological ligands within templates would have changed since CASP8 (Mike Tress pers. comm.), there was a small chance the list of relevant ligands in FireDB could have been influenced by new related PDB entries since CASP8. In addition, during the CASP9 prediction season our prototype version of the server often had connectivity problems with the database. In order to become independent of querying the FireDB database, a list of biologically relevant ligands was obtained from the Sternberg group, which was originally used by the 3D LigandSite server [15]. Using this list allows the FunFOLD standalone software and the current version of the FunFOLD server to be more reliable, independent on external servers and suitable for benchmarking on both of the CASP datasets.

The FunFOLD algorithm used the TM-align method [29] to superpose each of the template structures containing relevant ligands onto the 3D protein model. Each model-to-template superposition was saved if the resulting TM-score ≥0.4 (TM-scores ~0.4-0.6 have been shown to mark the transition phase of significantly related folds [31]). A simple PyMOL script allowed each of the superposition files to be used in order to orientate the original PDB structures with bound ligands correctly relative to the model. The resulting PyMOL superposition of all templates and models was then saved and parsed to leave only the coordinates of the model and relevant ligands.

Ligands were then assigned to clusters using an agglomerative hierarchical clustering algorithm that identified each continuous mass of contacting ligands, thereby indicating putative binding pockets. Ligands were considered to be part of a cluster if any of their atoms were in contact with the continuous mass. Thus, the linkage criteria for clustering were determined by contacts between ligands, which were defined as ≤ the Van der Waals radius of an atom plus 0.5 Ångströms. Once each continuous mass of contacting ligands was identified, the cluster with the largest number of ligands was selected as the location of the most likely binding pocket. The distances between all atoms within the mass of ligands and all atoms within the 3D protein model were then calculated. Again, residues were determined to be in contact with the ligand if the distance between a ligand atom and a residue atom was ≤ the Van der Waals radius plus 0.5 Ångströms.

In order to determine which residues were most likely to bind to the predicted ligand, a "residue voting" procedure was carried out. For a residue to be included in a prediction it must have had at least one contact with 2 or more ligands and at least 25% of the ligands in the cluster. This cut-off was determined during the CASP9 prediction season whilst the method was in development and was based on comparisons of the FunFOLD server output with that obtained from state-of-the-art servers, such as 3D LigandSite, that were publicly available at the time. However, no optimization of this cut-off was carried out prior to testing of the FunFOLD software on the CASP8 and CASP9 data sets.

**Figure 1:**
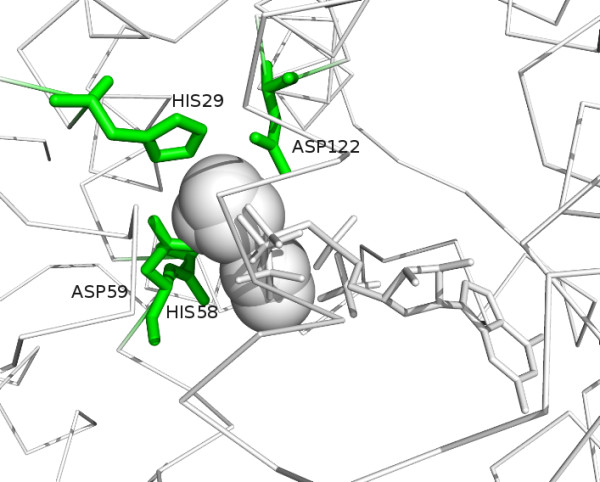
**The FunFOLD residue voting system**. Ribbon diagram of CASP8 target T0470 (PDBID 3djb) which illustrates how the voting system works in FunFOLD. The green sticks represented predicted ligand binding residues (which are the same as the observed ligand binding residues) and the white spheres and sticks represent predicted ligands.

The residue voting system used in FunFOLD can be illustrated in Figure 1. In Figure 1 we can see a cluster that contains a continuous mass of metals (1 ZN, 6 FEs, 1 NI and 1 MG), 1 GDP and 3 PO_4_s. The residues that are only contacting the GDP ligand are not considered as potential ligand binding residues, as the residues only receive 1/13 votes each (7.69%). Whilst the residues that are in contact with GDP and one PO_4 _molecule receive 2 votes, they only receive a 15% share of the vote (2/13) and so they are also excluded from the prediction. The final predicted ligand binding residues are therefore: HIS29 with 7/13 votes (53.85%), HIS58 with 11/13 votes (84.62%), ASP59 with 12/13 votes (92.31%) and ASP122 with 5/13 votes (38.46%); all are above the 25% threshold required to be included in the prediction.

### Accuracy Benchmarking

The FunFOLD method was benchmarked using only the information concerning templates and models for each target that could be obtained from the CASP8 and CASP9 server predictions. Thus all of the information used was available to predictors during both CASP prediction seasons. The FN prediction files for the 27 targets analysed for function prediction in CASP8 [1], the 30 targets analysed for function prediction in CASP9, and all associated 3D server models were downloaded from the CASP website [32].

For each CASP8 target, the ModFOLDclust2 method [28] was used to select the top 3D model from the server models submitted at CASP8. However, the top model from the IntFOLD-TS server (TS275) [33] was used for testing performance on each of the CASP9 targets. The IntFOLD-TS models were used so that the method is the same as the current FunFOLD server implementation. The top model for each target was then used as the starting model for predicting ligand binding residues. The parent records from each server model were examined in order to construct a list of template PDB IDs for each target that was available at the time of each CASP prediction season. The resulting template list was then filtered using FASTA [34] to ensure it was 70% non-redundant according to pairwise sequence identity. This type of filtering is in line with that carried out during the construction of the non-redundant fold libraries used by many fold recognition servers, such as IntFOLD-TS. Finally, a maximum of 40 templates were used in our analysis for efficiency.

The FunFOLD prediction results were compared against all of the function prediction groups participating in CASP8 and CASP9 using the MCC scores [35] as an indicator of performance. An analysis of the statistical significance between the differences in mean scores was also carried out, similar to that of the official CASP assessments [1,27]. In addition, a new metric, the Binding-site Distance Test (BDT) score [36] was used with the d_0 _threshold set to 1Ǻ, in order to stringently assess the accuracy of predictions. The BDT score ranges between 0 (random) and 1 (perfect) and relates to the actual 3D distance between the predicted residues and the observed residues. The BDT score appropriately penalizes both under and over predictions, whilst also considering the 3D distance of predicted residues from the observed binding site. Thus, predictions close to the binding site score higher than more distant predictions [36].

The top function prediction methods in CASP8 were methods by the Lee group (FN407 & FN293) [3] and the Sternberg group (FN202)[22]. In order to more stringently compare the performance of the FunFOLD method against that of the Lee and Sternberg methods, for each prediction we also used the top 3D models that the Lee and the Sternberg groups submitted for their CASP8 server and manual predictions (LEE-S_TS1, LEE_TS1 and Phyre-de-novo_TS1). This analysis was also carried out on the CASP9 data set, using models from both the IntFOLD-TS server (FN275) and the Zhang-server (FN428) methods. This allowed us to gauge how much of the difference in performance was due to the initial model selection. Finally, the analysis was repeated using native structures for each CASP target in order to evaluate performance using "perfect models".

## Results

The FunFOLD method for the prediction of ligand binding residues is benchmarked using the set of 27 CASP8 function prediction targets and the set of 30 CASP9 function prediction targets. The CASP sets are further subdivided according to the types of ligand bound, as below:

CASP8 targets: Metal - T0391, T0406, T0407, T0410, T0425, T0426, T0440, T0444, T0453, T0457, T0461, T0470, T0476, T0478, T0480, T0487; Non-metal - T0394, T0396, T0422, T0430, T0431, T0450, T0477, T0483, T0485, T0490, T0508

CASP9 targets: Metal - T0518, T0521, T0529, T0539, T0548, T0585, T0625, T0629, T0635; Non-metal - T0515, T0516, T0524, T0526, T0547, T0565, T0570, T0582, T0584, T0591, T0597, T0599, T0604, T0607, T0609, T0613, T0615, T0622, T0632, T0636, T0641

(In CASP8 some targets had both metals and non-metals, but these were defined by the assessors as non-metals. For consistency, we do the same with the CASP9 targets.)

The performance of FunFOLD is compared against that of groups that participated in the CASP8 and CASP9 function prediction categories.

### Accuracy Benchmarking

**Figure 2:**
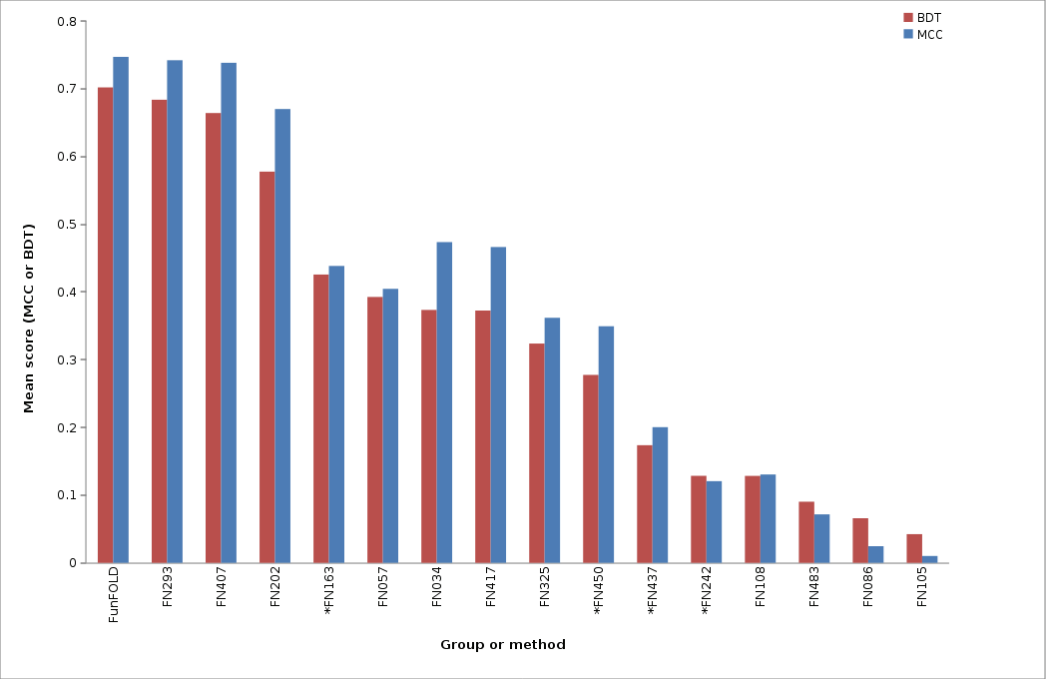
**MCC scores and BDT scores for CASP8 benchmarking**. Mean per-target MCC scores and BDT scores for each CASP8 function prediction group and the FunFOLD method (minimum of 15 predictions). * indicates server methods.

**Figure 3:**
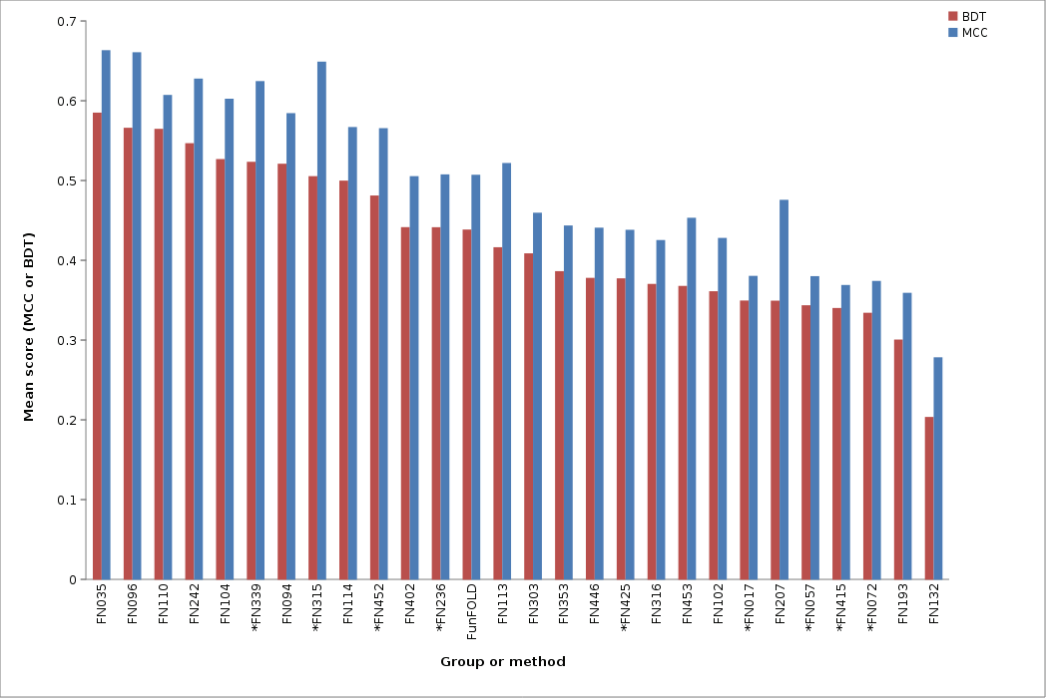
**MCC scores and BDT scores for CASP9 benchmarking (partial binding site definition)**. Mean per-target MCC scores and BDT scores for each CASP9 function prediction group and the FunFOLD method (minimum of 15 predictions). * indicates server methods.

**Figure 4:**
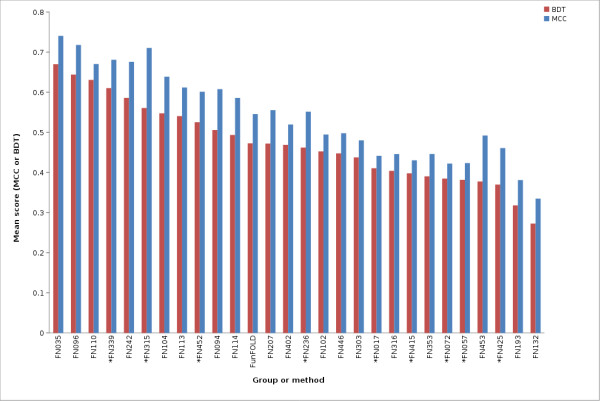
**MCC scores and BDT scores for CASP9 benchmarking (extended binding site definition)**. Mean per-target MCC scores and BDT scores for each CASP9 function prediction group and the FunFOLD method (minimum of 15 predictions). * indicates server methods.

**Figure 5:**
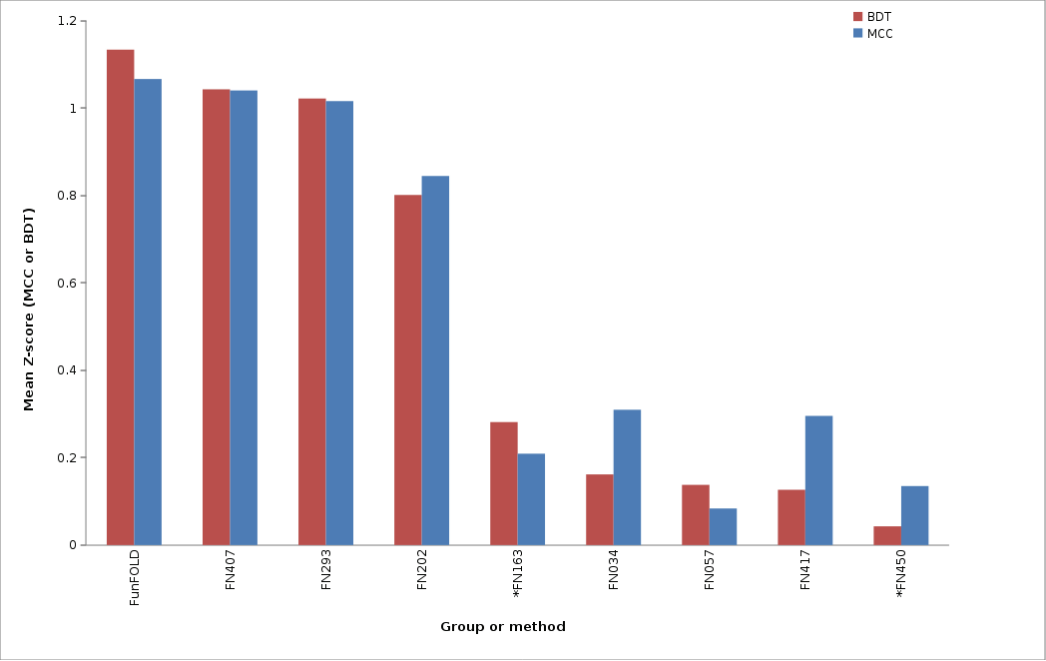
**MCC Z-scores and BDT Z-scores for CASP8 benchmarking**. Mean per-target MCC Z-scores and BDT Z-scores for each CASP8 function prediction group and the FunFOLD method (minimum of 15 predictions; only groups with positive Z-scores are shown). * indicates server methods.

**Figure 6:**
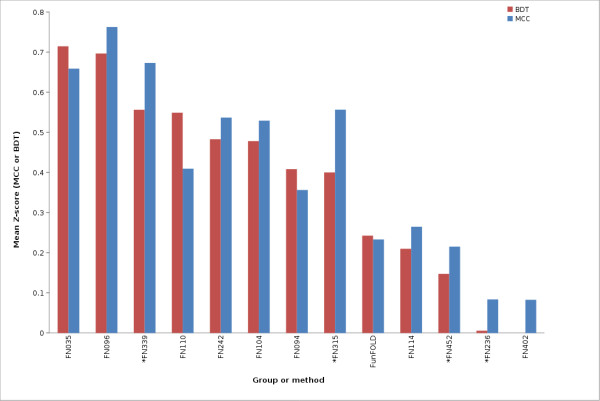
**MCC Z-scores and BDT Z-scores for CASP9 benchmarking (partial binding site definition)**. Mean per-target MCC Z-scores and BDT Z-scores for each CASP9 function prediction group and the FunFOLD method (minimum of 15 predictions; only groups with positive Z-scores are shown). * indicates server methods.

**Figure 7:**
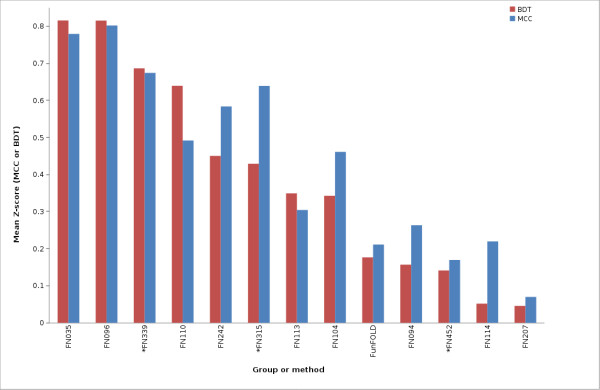
**MCC Z-scores and BDT Z-scores for CASP9 benchmarking (extended binding site definition)**. Mean per-target MCC Z-scores and BDT Z-scores for each CASP9 function prediction group and the FunFOLD method (minimum of 15 predictions; only groups with positive Z-scores are shown). * indicates server methods.

The results of an assessment of binding site predictions, similar to the official CASP8 function prediction assessment carried out by Lopez et al. (2009) [1], are shown in Figure 2, 3, 4, 5, 6 and 7 and in all tables. The Matthews Correlation Coefficient (MCC) and the Binding-site Distance Test (BDT) method are used to measure prediction success and the resulting scores achieved by the different groups are compared with those from the FunFOLD method. The FunFOLD method is shown to outperform all other methods tested at CASP8 according to both the mean per-target MCC scores and BDT scores (Figure 2).

The FunFOLD method is also shown to be competitive with the methods tested at CASP9. The FunFOLD method outperformed the 3DLigandSite methods (FN017, FN415, FN057, FN072), but did not outperform the top server methods, I-TASSER-FUNCTION (FN339) and firestar (FN315), according to mean per-target MCC and BDT scores for both partial and extended binding site analysis (Figure 3 and Figure 4). (The CASP9 assessors defined partial binding site residues as binding site residues from CASP9 targets that contain ligands, with extended binding sites referring to CASP9 targets that bound ligands that were not the target's natural ligand, thus, the natural ligand was docked into the binding site and the binding site residues predicted.).

The mean MCC Z-scores and BDT Z-scores were also calculated for each group in order to normalize scores across targets and the results are shown in Figure 5, Figure 6 and Figure 7. Again, the predictions from the FunFOLD method are shown to outperform those from all of the other CASP8 groups (Figure 5) and outperformed all server methods tested at CASP9 with the exception of I-TASSER-FUNCTION (FN339) and firestar (FN315) (Figures 6 and 7).

Whilst the results shown in Figures 2, 3, 4, 5, 6 and 7 indicate that the FunFOLD method is competitive, each group has made predictions for different numbers of targets. Therefore, a direct comparison of methods from CASP8 and CASP9 on common subsets of targets must be carried out along with an analysis of statistical significance of the differences in performance. The results in Table 1 show that the FunFOLD method does achieve higher mean scores and mean Z-scores than every other CASP8 method, when common subsets of predictions are directly compared.

**Table 1:** The mean MCC and BDT raw scores and Z-scores obtained by FunFOLD are compared with those obtained by each CASP8 function prediction group.

Group ID	N	Mean score for group		Mean score for FunFOLD		Increase in mean score		Mean Z-score for group		Mean Z-score for FunFOLD		P-value (raw score)		P-value (Z-score)		1 - p-value (raw score)		1 - p-value (Z-score)	
		
		MCC	BDT	MCC	BDT	MCC	BDT	MCC	BDT	MCC	BDT	MCC	BDT	MCC	BDT	MCC	BDT	MCC	BDT
FN407	25	0.768	0.689	**0.778**	**0.702**	0.010	0.013	1.105	1.099	**1.137**	**1.134**	0.734	0.487	0.699	0.526	0.266	0.513	0.301	0.474
FN293	19	0.742	0.684	**0.789**	**0.748**	0.050	0.064	1.016	1.022	**1.164**	**1.254**	0.318	0.128	0.285	0.172	0.682	0.872	0.715	0.828
FN202	23	0.670	0.578	**0.797**	**0.754**	0.130	0.176	0.845	0.801	**1.158**	**1.259**	**0.004**	**0.001**	**0.006**	**0.004**	0.996	0.999	0.994	0.996
FN417	26	0.485	0.387	**0.747**	**0.702**	0.260	0.315	0.335	0.165	**1.066**	**1.134**	**0.000**	**0.000**	**0.000**	**0.000**	1.000	1.000	1.000	1.000
FN034	23	0.488	0.387	**0.810**	**0.762**	0.320	0.375	0.249	0.086	**1.200**	**1.283**	**0.000**	**0.000**	**0.000**	**0.000**	1.000	1.000	1.000	1.000
FN209	11	0.465	0.376	**0.779**	**0.694**	0.310	0.319	0.230	0.163	**1.200**	**1.207**	**0.000**	**0.000**	**0.000**	**0.000**	1.000	1.000	1.000	1.000
FN163	23	0.438	0.426	**0.717**	**0.668**	0.280	0.242	0.209	0.281	**1.017**	**1.066**	**0.000**	**0.002**	**0.000**	**0.002**	1.000	0.998	1.000	0.998
FN057	24	0.405	0.393	**0.769**	**0.718**	0.360	0.326	0.084	0.138	**1.118**	**1.184**	**0.000**	**0.000**	**0.000**	**0.000**	1.000	1.000	1.000	1.000
FN450	25	0.358	0.286	**0.755**	**0.715**	0.400	0.429	0.073	-0.031	**1.072**	**1.162**	**0.000**	**0.000**	**0.000**	**0.001**	1.000	1.000	1.000	0.999
FN325	25	0.377	0.336	**0.778**	**0.730**	0.400	0.394	-0.003	-0.015	**1.131**	**1.203**	**0.000**	**0.000**	**0.000**	**0.000**	1.000	1.000	1.000	1.000
FN437	17	0.209	0.183	**0.768**	**0.716**	0.560	0.534	-0.564	-0.603	**1.151**	**1.243**	**0.000**	**0.000**	**0.000**	**0.000**	1.000	1.000	1.000	1.000
FN108	24	0.136	0.133	**0.769**	**0.730**	0.630	0.597	-0.682	-0.663	**1.121**	**1.203**	**0.000**	**0.000**	**0.000**	**0.000**	1.000	1.000	1.000	1.000
FN483	21	0.076	0.094	**0.723**	**0.669**	0.650	0.575	-0.736	-0.719	**1.040**	**1.063**	**0.000**	**0.000**	**0.000**	**0.000**	1.000	1.000	1.000	1.000
FN242	26	0.126	0.133	**0.747**	**0.702**	0.620	0.569	-0.744	-0.704	**1.066**	**1.134**	**0.000**	**0.000**	**0.000**	**0.000**	1.000	1.000	1.000	1.000
FN105	26	0.011	0.044	**0.747**	**0.702**	0.740	0.658	-1.069	-0.983	**1.066**	**1.134**	**0.000**	**0.000**	**0.000**	**0.000**	1.000	1.000	1.000	1.000
FN086	24	0.024	0.068	**0.781**	**0.728**	0.760	0.660	-1.077	-0.933	**1.151**	**1.222**	**0.000**	**0.000**	**0.000**	**0.000**	1.000	1.000	1.000	1.000

The analysis is based on common subsets of *all *CASP8 function prediction targets, with a minimum of 10 predictions in common. N, size of the common subset used in the comparison; MCC, Matthews Correlation Coefficient; BDT, the Binding Site Distance Test Score [36].P-value, the p-value for the paired Wilcoxon signed rank sum test using the raw scores; P-value (Z-score), the p-value for the paired Wilcoxon signed rank sum test using the Z-scores; 1 - p-value, 1 minus the p-value for the paired Wilcoxon signed rank sum test using the raw scores; 1-p-value (Z-score), 1 minus the p-value for the paired Wilcoxon signed rank sum test using the Z-scores. The tables are sorted by the Mean MCC Z-score for groups with the best CASP8 groups at the top. The highest mean scores, mean Z-scores, significant p-values and significant 1-p-values are indicated in bold. Server groups are underlined (FN293 and FN105 were extended deadline server groups; no publicly available server could be found for FN293 at the time of writing; group FN202 now have a publicly available server which is comparable to their CASP8 performance [15]).

The difference in mean MCC performance is >28% for each of the true server methods tested at CASP8. In addition, the FunFOLD method shows a 13% improvement over group FN202's CASP8 predictions, a 5% improvement over group FN293's CASP8 predictions and a 1% improvement over FN407's CASP8 predictions. The improvement is statistically significant for all CASP8 groups tested, except the methods by the Lee group - FN407 and FN293. A similar increase in performance is shown if the BDT score is used for the assessment (Table 1).

Tables 2 and 3 show the performance of methods on the subsets of targets; those containing only metal ligands (Table 2) and those containing non-metal ligands (Table 3). On the CASP8 data set the FunFOLD method performs slightly better on the subset of targets containing metal ligands than those containing non-metals. The FunFOLD method does improve upon the majority of other methods when tested on the non-metal subset; however there is no significant difference compared with the top four methods (Table 3).

**Table 2:** The mean MCC and BDT raw scores and Z-scores obtained by FunFOLD are compared with those obtained by each CASP8 function prediction group - as in Table 1 except for CASP8 targets containing *only metal ligands*.

Group ID	N	Mean score for group		Mean score for FunFOLD		Increase in mean score		Mean Z-score for group		Mean Z-score for FunFOLD		P-value (raw score)		P-value (Z-score)		1 - p-value (raw score)		1 - p-value (Z-score)	
		
		MCC	BDT	MCC	BDT	MCC	BDT	MCC	BDT	MCC	BDT	MCC	BDT	MCC	BDT	MCC	BDT	MCC	BDT
FN407	14	0.764	0.981	**0.799**	**0.710**	0.035	0.029	1.064	1.099	**1.197**	**1.218**	0.541	0.447	0.541	0.553	0.459	0.553	0.459	0.447
FN202	14	0.654	0.582	**0.798**	**0.761**	0.145	0.233	0.869	0.803	**1.185**	**1.349**	**0.025**	**0.009**	**0.047**	**0.035**	0.975	0.991	0.953	0.965
FN034	13	0.620	0.460	**0.862**	**0.819**	0.241	0.359	0.641	0.430	**1.330**	**1.522**	**0.007**	**0.002**	**0.005**	**0.002**	0.993	0.998	0.995	0.998
FN417	15	0.143	0.283	**0.744**	**0.710**	0.331	0.427	0.183	-0.050	**1.070**	**1.218**	**0.002**	**0.001**	**0.004**	**0.001**	0.998	0.999	0.996	0.999
FN450	15	0.302	0.198	**0.744**	**0.710**	0.441	0.512	0.070	-0.077	**1.070**	**1.218**	**0.000**	**0.000**	**0.028**	**0.032**	1.000	1.000	0.972	0.968
FN325	14	0.367	0.318	**0.798**	**0.761**	0.432	0.443	0.055	0.052	**1.185**	**1.349**	**0.002**	**0.002**	**0.005**	**0.005**	0.998	0.998	0.995	0.995
FN057	13	0.201	0.189	**0.783**	**0.742**	0.582	0.553	-0.470	-0.446	**1.165**	**1.324**	**0.000**	**0.000**	**0.000**	**0.000**	1.000	1.000	1.000	1.000
FN163	12	0.178	0.169	**0.685**	**0.647**	0.507	0.478	-0.485	-0.444	**0.976**	**1.110**	**0.000**	**0.001**	**0.000**	**0.001**	1.000	0.999	1.000	0.999
FN483	12	0.066	0.071	**0.699**	**0.656**	0.633	0.585	-0.546	-0.552	**1.023**	**1.116**	**0.001**	**0.001**	**0.008**	**0.002**	0.999	0.999	0.992	0.998
FN108	13	0.119	0.092	**0.783**	**0.761**	0.664	0.669	-0.566	-0.594	**1.172**	**1.349**	**0.000**	**0.000**	**0.001**	**0.001**	1.000	1.000	0.999	0.999
FN242	15	0.138	0.157	**0.744**	**0.710**	0.609	0.554	-0.583	-0.437	**1.070**	**1.218**	**0.000**	**0.000**	**0.000**	**0.000**	1.000	1.000	1.000	1.000
FN105	15	0.004	0.030	**0.744**	**0.710**	0.739	0.680	-0.929	-0.818	**1.070**	**1.218**	**0.000**	**0.000**	**0.000**	**0.000**	1.000	1.000	1.000	1.000
FN086	13	-0.017	0.025	**0.805**	**0.760**	0.822	0.735	-1.092	-0.884	**1.228**	**1.394**	**0.000**	**0.000**	**0.000**	**0.000**	1.000	1.000	1.000	1.000

**Table 3:** The mean MCC and BDT raw scores and Z-scores obtained by FunFOLD are compared with those obtained by each CASP8 function prediction group - as in Table 1 except for CASP8 targets containing *non-metal ligands *or *metal and non-metal ligands *in the same binding site.

Group ID	N	Mean score for group		Mean score for FunFOLD		Increase in mean score		Mean Z-score for group		Mean Z-score for FunFOLD		P-value (raw score)		P-value (Z-score)		1 - p-value (raw score)		1 - p-value (Z-score)	
		
		MCC	BDT	MCC	BDT	MCC	BDT	MCC	BDT	MCC	BDT	MCC	BDT	MCC	BDT	MCC	BDT	MCC	BDT
FN407	11	**0.774**	**0.700**	0.752	0.690	-0.022	-0.010	**1.156**	**1.100**	1.061	1.018	0.681	0.517	0.681	0.449	0.319	0.483	0.319	0.551
FN293	10	**0.778**	0.703	0.771	**0.722**	-0.006	0.020	**1.101**	0.008	1.074	**1.077**	0.423	0.278	0.423	0.313	0.577	0.722	0.577	0.688
FN163	11	0.723	**0.705**	**0.752**	0.690	0.029	-0.015	0.966	**1.072**	**1.061**	1.018	0.183	0.382	0.183	0.416	0.817	0.618	0.817	0.584
FN057	11	0.646	0.633	**0.752**	**0.690**	0.160	0.058	0.738	0.828	**1.061**	**1.018**	0.139	0.382	0.120	0.381	0.861	0.618	0.880	0.618
FN417	11	0.583	0.528	**0.752**	**0.690**	0.169	0.162	0.541	0.458	**1.061**	**1.018**	**0.012**	**0.021**	**0.012**	**0.042**	0.988	0.979	0.988	0.958
FN450	10	0.442	0.418	**0.771**	**0.722**	0.330	0.305	0.077	0.038	**1.074**	**1.077**	**0.002**	**0.003**	**0.002**	**0.003**	0.988	0.997	0.998	0.997
FN325	11	0.389	0.360	**0.752**	**0.690**	0.362	0.331	-0.076	-0.102	**1.061**	**1.018**	**0.003**	**0.002**	**0.003**	**0.002**	0.987	0.998	0.997	0.998
FN034	10	0.317	0.292	**0.743**	**0.687**	0.426	0.395	-0.026	-0.362	**1.031**	**0.972**	**0.003**	**0.005**	**0.006**	**0.005**	0.994	0.995	0.994	0.995
FN108	11	0.156	0.185	**0.752**	**0.690**	0.596	0.505	-0.819	-0.750	**1.061**	**1.018**	**0.000**	**0.000**	**0.000**	**0.000**	1.000	1.000	1.000	1.000
FN242	11	0.109	0.101	**0.752**	**0.690**	0.643	0.590	-0.965	-1.069	**1.061**	**1.018**	**0.000**	**0.000**	**0.000**	**0.000**	1.000	1.000	1.000	1.000
FN086	11	0.074	0.118	**0.752**	**0.690**	0.678	0.572	-1.061	-0.990	**1.061**	**1.018**	**0.000**	**0.000**	**0.000**	**0.000**	1.000	1.000	1.000	1.000
FN105	11	0.020	0.063	**0.752**	**0.690**	0.731	0.628	-1.260	-1.207	**1.061**	**1.018**	**0.000**	**0.000**	**0.000**	**0.000**	1.000	1.000	1.000	1.000

In order to produce the FunFOLD results shown in Table 1, 2 and 3, the ModFOLDclust2 quality assessment program [28] was used to select a 3D model for each target and this model was then used as an input for the FunFOLD executable. However, the results in Table 4 are also shown in order to compare the performance of FunFOLD if the TS1 3D models submitted by each CASP8 comparison group are used instead. An improvement in MCC score of around 10% is shown compared to group FN202's predictions, which again is shown to be statistically significant. The improvement over group FN293's predictions is ~3%, whilst the improvement over group FN407 is increased to ~2%. Again, using the BDT score shows that a significant increase over group FN202 is maintained along with an increase in mean scores compared with those obtained by groups FN407 and FN293. In Table 5, the coordinates from the native structures are used in order to determine the difference in performance compared with the top 3 groups. Using native structures instead of models, the FunFOLD method shows a significant improvement over both groups FN293 and FN202.

**Table 4:** What is the effect on FunFOLD performance if different starting models are used?

Group ID	N	Mean score for group		Mean score for FunFOLD		Increase in mean score		Mean Z-score for group		Mean Z-score for FunFOLD		P-value (raw score)		P-value (Z-scores)		1 - p-value (raw score)		1 - p-value (Z-score)	
		
		MCC	BDT	MCC	BDT	MCC	BDT	MCC	BDT	MCC	BDT	MCC	BDT	MCC	BDT	MCC	BDT	MCC	BDT
FN407	25	0.768	0.689	**0.789**	**0.697**	0.021	0.008	1.097	1.095	**1.169**	**1.128**	0.493	0.528	0.478	0.542	0.507	0.472	0.522	0.458
FN293	19	0.742	0.684	**0.774**	**0.714**	0.032	0.030	1.017	1.027	**1.134**	**1.158**	0.370	0.269	0.301	0.301	0.630	0.731	0.699	0.699
FN202	22	0.692	0.593	**0.791**	**0.716**	0.098	0.123	0.868	0.824	**1.142**	**1.183**	**0.015**	**0.027**	**0.018**	**0.040**	0.985	0.973	0.982	0.960

A repeat comparison of FunFOLD against the top 3 groups at CASP8, using TS1 3D models obtained from each comparison group. LEE_TS1 models were used for the FunFOLD comparison with group FN407, LEE-S_TS1 models for the comparison with FN293 and Phyre-de-novo_TS1 for the comparison with FN202.

**Table 5:** What is the effect on FunFOLD performance if the native structures are used for each target?

Group ID	N	Mean score for group		Mean score for FunFOLD		Increase in mean score		Mean Z-score for group		Mean Z-score for FunFOLD		P-value (raw score)		P-value (Z-score)		1 - p-value (raw score)		1 - p-value (Z-score)	
		
		MCC	BDT	MCC	BDT	MCC	BDT	MCC	BDT	MCC	BDT	MCC	BDT	MCC	BDT	MCC	BDT	MCC	BDT
FN407	23	0.793	0.735	**0.853**	**0.774**	0.060	0.039	1.160	1.238	**1.343**	**1.388**	0.112	0.255	0.119	0.277	0.888	0.746	0.881	0.723
FN293	18	0.786	0.721	**0.891**	**0.828**	0.106	0.107	1.086	1.091	**1.413**	**1.483**	**0.009**	**0.330**	**0.008**	**0.033**	0.991	0.967	0.991	0.967
FN202	21	0.691	0.593	**0.863**	**0.790**	0.172	0.196	0.841	0.789	**1.334**	**1.414**	**0.001**	**0.001**	**0.001**	**0.001**	0.999	0.999	0.999	0.999

A repeat comparison of FunFOLD performance versus the top 3 groups at CASP8.

In order to produce the FunFOLD results shown in Tables 6, 7, 8, 9, 10 and 11, the top IntFOLD-TS model for each target was used as an input for the FunFOLD executable. This was done so that the method matched the current implementation of the FunFOLD server. The results indicate that there is no statistically significant difference between the top server methods tested at CASP9 and the FunFOLD method, according to p-values of the raw and z-scores, on the partial (Table 6, 7 and 8) and extended binding sites (Tables 9, 10 and 11). Again, the CASP9 dataset is divided into subsets of targets containing metal and non-metal ligands and the results are shown in Tables 7, 8, 10 and 11. However, there are no notable differences compared with using the full data (Tables 6 and 9).The results actually show fewer significant differences between methods, which is expected due to the reduced sizes of the subsets (Tables 7, 8, 10 and 11).

**Table 6:** The mean MCC and BDT raw scores and Z-scores obtained by FunFOLD are compared with those obtained by the top CASP9 function prediction groups for the partial binding sites analysis.

Group ID	N	Mean score for group		Mean score for FunFOLD		Difference in mean score		Mean Z-score for group		Mean Z-score for FunFOLD		P-value (raw score)		P-value (Z-score)		1 - p-value (raw score)		1 - p-value (Z-score)	
		
		MCC	BDT	MCC	BDT	MCC	BDT	MCC	BDT	MCC	BDT	MCC	BDT	MCC	BDT	MCC	BDT	MCC	BDT
FN096	24	**0.626**	**0.520**	0.508	0.439	-0.188	-0.018	**0.526**	**0.413**	0.052	0.108	0.954	0.879	0.938	0.853	**0.004**	**0.049**	0.062	**0.049**
FN035	20	**0.636**	**0.545**	0.531	0.467	-0.105	-0.078	**0.512**	**0.593**	0.095	0.144	0.897	0.951	0.904	0.951	0.103	0.131	0.096	0.241
FN339	24	**0.597**	**0.492**	0.508	0.439	-0.089	-0.053	**0.458**	**0.333**	0.052	0.108	0.932	0.772	0.897	0.815	0.068	0.121	0.103	0.147
FN242	23	**0.591**	**0.505**	0.504	0.439	-0.087	-0.066	**0.457**	**0.404**	0.023	0.101	0.948	0.767	0.952	0.817	0.052	0.233	**0.048**	0.183
FN104	22	**0.619**	**0.538**	0.513	0.447	-0.106	-0.091	**0.366**	**0.350**	0.004	0.043	0.973	0.962	0.968	0.912	**0.027**	**0.038**	**0.032**	0.088
FN315	21	**0.620**	**0.459**	0.538	0.439	-0.081	-0.020	**0.332**	**0.152**	0.085	0.108	0.784	0.698	0.671	0.637	0.216	0.228	0.329	0.185
FN110	22	**0.594**	**0.548**	0.546	0.470	-0.048	-0.078	**0.233**	**0.430**	0.133	0.161	0.487	0.869	0.375	0.759	0.513	0.277	0.625	0.364
FN094	23	**0.552**	**0.482**	0.531	0.458	-0.021	-0.024	**0.171**	**0.177**	0.129	0.168	0.596	0.723	0.537	0.636	0.404	0.302	0.463	0.363
FN114	23	**0.548**	**0.479**	0.499	0.436	-0.049	-0.044	**0.063**	0.014	0.005	**0.055**	0.745	0.723	0.555	0.458	0.255	0.277	0.445	0.542
FN452	22	**0.543**	0.448	0.518	**0.451**	-0.025	0.003	0.011	-0.082	**0.023**	**0.091**	0.588	0.334	0.524	0.257	0.412	0.666	0.476	0.743
FN236	24	0.486	0.418	**0.508**	**0.439**	0.022	0.021	-0.049	-0.147	**0.052**	**0.108**	0.440	0.361	0.404	0.269	0.596	0.639	0.596	0.731
FN425	23	0.409	0.346	**0.507**	**0.439**	0.097	0.093	-0.323	-0.349	**0.035**	**0.111**	0.111	**0.031**	0.111	0.054	0.886	0.969	0.889	0.946
FN017	23	0.403	0.376	**0.531**	**0.458**	0.128	0.094	-0.501	-0.397	**0.129**	**0.168**	**0.019**	**0.016**	**0.013**	**0.011**	0.981	0.941	0.987	0.951
FN057	23	0.424	0.364	**0.531**	**0.458**	0.107	0.082	-0.534	-0.373	**0.129**	**0.168**	**0.026**	0.059	**0.013**	**0.049**	0.974	0.984	0.987	0.989
FN113	22	0.477	0.381	**0.523**	**0.456**	0.046	0.075	-0.548	-0.594	**0.082**	**0.114**	0.126	**0.049**	**0.044**	**0.035**	0.874	0.972	0.956	0.984
FN072	23	0.418	0.366	**0.531**	**0.458**	0.113	0.092	-0.551	-0.446	**0.129**	**0.168**	**0.019**	**0.028**	**0.012**	**0.016**	0.981	0.991	0.988	0.996
FN415	23	0.388	0.353	**0.531**	**0.458**	0.142	0.105	-0.619	-0.542	**0.129**	**0.168**	**0.007**	**0.009**	**0.006**	**0.004**	0.993	0.951	0.994	0.975

The analysis is based on common subsets of *all *CASP9 targets, with a minimum of 10 predictions in common. N, size of the common subset used in the comparison; MCC, Matthews Correlation Coefficient; BDT, the Binding Site Distance Test Score [36].P-value, the p-value for the paired Wilcoxon signed rank sum test using the raw scores; P-value (Z-score), the p-value for the paired Wilcoxon signed rank sum test using the Z-scores; 1 - p-value, 1 minus the p-value for the paired Wilcoxon signed rank sum test using the raw scores; 1-p-value (Z-score), 1 minus the p-value for the paired Wilcoxon signed rank sum test using the Z-scores. The table is sorted by the Mean MCC Z-score for groups with the best CASP9 groups at the top. The highest mean scores, mean Z-scores, significant p-values and significant 1-p-values are indicated in bold. Server groups are underlined.

**Table 7:** The mean MCC and BDT raw scores and Z-scores obtained by FunFOLD are compared with those obtained by the top CASP9 function prediction groups - as in Table 6 except for CASP9 targets containing *only metal ligands*.

Group ID	N	Mean score for group		Mean score for FunFOLD		Difference in mean score		Mean Z-score for group		Mean Z-score for FunFOLD		P-value (raw score)		P-value (Z-score)		1 - p-value (raw score)		1 - p-value (Z-score)	
		
		MCC	BDT	MCC	BDT	MCC	BDT	MCC	BDT	MCC	BDT	MCC	BDT	MCC	BDT	MCC	BDT	MCC	BDT
FN114	6	**0.642**	**0.560**	0.408	0.343	-0.234	-0.217	**0.602**	0.777	-0.264	-0.275	0.947	0.970	0.911	0.947	0.053	**0.030**	0.089	0.053
FN094	6	**0.651**	**0.521**	0.530	0.428	-0.122	-0.092	**0.555**	0.551	0.210	0.159	0.860	0.860	0.791	0.860	0.140	0.140	0.209	0.140
FN452	6	**0.626**	**0.518**	0.492	0.397	-0.134	-0.121	**0.417**	0.491	-0.079	-0.104	0.911	0.860	0.911	0.911	0.089	0.140	0.089	0.089
FN242	7	**0.540**	**0.438**	0.450	0.367	-0.089	-0.071	**0.381**	0.369	-0.063	-0.044	0.911	0.735	0.911	0.799	0.089	0.265	0.089	0.201
FN339	7	**0.536**	**0.424**	0.450	0.367	-0.085	-0.057	**0.345**	0.406	-0.063	-0.044	0.853	0.735	0.799	0.896	0.147	0.265	0.201	0.104
FN096	7	**0.562**	**0.420**	0.450	0.367	-0.112	-0.053	**0.258**	0.207	-0.063	-0.044	0.853	0.663	0.735	0.853	0.147	0.337	0.265	0.147
FN236	7	**0.470**	**0.369**	0.450	0.367	-0.019	-0.002	**-0.009**	-0.065	-0.063	**-0.044**	0.606	0.606	0.606	0.500	0.394	0.394	0.394	0.500
FN057	6	0.371	0.250	**0.530**	**0.428**	0.158	0.178	-0.565	-0.770	**0.210**	**0.159**	0.109	0.031	0.156	**0.031**	0.891	0.969	0.844	0.969
FN072	6	0.360	0.239	**0.530**	**0.428**	0.170	0.189	-0.571	-0.817	**0.210**	**0.159**	0.109	0.047	0.156	**0.031**	0.891	0.953	0.844	0.969
FN425	7	0.281	0.226	**0.450**	**0.367**	0.169	0.142	-0.626	-0.546	**-0.063**	**-0.044**	0.209	0.089	0.209	0.140	0.791	0.911	0.791	0.860
FN017	6	0.233	0.201	**0.530**	**0.428**	0.297	0.227	-1.143	-1.008	**0.210**	**0.159**	**0.031**	0.016	**0.031**	**0.016**	0.969	0.984	0.969	0.984
FN415	6	0.197	0.172	**0.530**	**0.428**	0.333	0.256	-1.356	-1.214	**0.210**	**0.159**	**0.031**	0.016	**0.031**	**0.016**	0.969	0.984	0.969	0.984

**Table 8:** The mean MCC and BDT raw scores and Z-scores obtained by FunFOLD are compared with those obtained by the top CASP9 function prediction groups - as in Table 6 except for CASP9 targets containing *non-metal ligands *or *metal and non-metal ligands *in the same binding site.

Group ID	N	Mean score for group		Mean score for FunFOLD		Difference in mean score		Mean Z-score for group		Mean Z-score for FunFOLD		P-value (raw score)		P-value (Z-score)		1 - p-value (raw score)		1 - p-value (Z-score)	
		
		MCC	BDT	MCC	BDT	MCC	BDT	MCC	BDT	MCC	BDT	MCC	BDT	MCC	BDT	MCC	BDT	MCC	BDT
FN096	18	**0.644**	**0.545**	0.536	0.465	-0.108	-0.081	**0.566**	**0.416**	0.140	0.194	0.941	0.848	0.877	0.710	0.059	0.152	0.123	0.290
FN339	18	**0.620**	**0.511**	0.536	0.465	-0.084	-0.046	**0.505**	**0.298**	0.140	0.194	0.790	0.551	0.776	0.517	0.210	0.449	0.224	0.483
FN035	18	**0.613**	**0.518**	0.536	0.465	-0.076	-0.053	**0.503**	**0.544**	0.140	0.194	0.888	0.909	0.888	0.909	0.112	0.091	0.112	0.091
FN242	17	**0.609**	**0.526**	0.533	0.467	-0.076	-0.059	**0.447**	**0.417**	0.106	0.188	0.789	0.510	0.803	0.612	0.211	0.490	0.197	0.388
FN315	18	**0.601**	**0.503**	0.536	0.465	-0.065	-0.038	**0.376**	**0.377**	0.140	0.194	0.710	0.779	0.665	0.752	0.290	0.221	0.335	0.248
FN104	18	**0.571**	**0.495**	0.536	0.465	-0.035	-0.030	**0.180**	0.192	0.140	**0.194**	0.776	0.710	0.746	0.567	0.224	0.290	0.254	0.433
FN110	18	**0.569**	**0.515**	0.536	0.465	-0.034	-0.050	**0.144**	**0.297**	0.140	0.194	0.416	0.617	0.290	0.500	0.584	0.383	0.710	0.500
FN094	18	0.526	**0.468**	**0.536**	0.465	0.010	-0.004	0.119	0.128	0.140	**0.194**	0.449	0.594	0.469	0.594	0.551	0.406	0.531	0.406
FN236	18	0.499	0.437	**0.536**	**0.465**	0.036	0.028	-0.017	-0.135	0.140	**0.194**	0.295	0.294	0.356	0.243	0.705	0.706	0.644	0.757
FN452	17	0.520	0.423	**0.533**	**0.467**	0.013	0.044	-0.065	-0.220	0.106	**0.188**	0.399	0.112	0.335	0.078	0.601	0.888	0.665	0.922
FN114	18	0.520	0.452	**0.536**	**0.465**	0.016	0.013	-0.092	-0.165	0.140	**0.194**	0.370	0.285	0.285	0.197	0.630	0.715	0.715	0.803
FN425	18	0.473	0.397	**0.536**	**0.467**	0.063	0.069	-0.150	-0.232	0.121	**0.203**	0.173	0.084	0.143	0.129	0.827	0.916	0.857	0.871
FN017	18	0.464	0.414	**0.536**	**0.465**	0.072	0.051	-0.303	-0.225	0.140	**0.194**	0.114	0.114	0.084	0.059	0.886	0.886	0.916	0.941
FN415	18	0.457	0.408	**0.536**	**0.465**	0.079	0.057	-0.401	-0.356	0.140	**0.194**	0.071	0.084	**0.049**	**0.030**	0.929	0.916	0.951	0.970
FN057	18	0.445	0.413	**0.536**	**0.465**	0.091	0.052	-0.538	-0.274	0.140	**0.194**	0.059	0.209	**0.017**	0.142	0.941	0.791	0.983	0.858
FN072	18	0.439	0.401	**0.536**	**0.465**	0.097	0.064	-0.590	-0.381	0.140	**0.194**	0.052	0.106	**0.016**	0.071	0.948	0.894	0.984	0.929
FN113	18	0.447	0.340	**0.536**	**0.465**	0.089	0.125	-0.810	-0.878	0.140	**0.194**	0.054	**0.003**	**0.012**	**0.003**	0.946	0.997	0.988	0.997

**Table 9:** The mean MCC and BDT raw scores and Z-scores obtained by FunFOLD are compared with those obtained by the top CASP9 function prediction groups for the extended binding sites analysis.

Group ID	N	Mean score for group		Mean score for FunFOLD		Difference in mean score		Mean Z-score for group		Mean Z-score for FunFOLD		P-value (raw score)		P-value (Z-score)		1 - p-value (raw score)		1 - p-value (Z-score)	
		
		MCC	BDT	MCC	BDT	MCC	BDT	MCC	BDT	MCC	BDT	MCC	BDT	MCC	BDT	MCC	BDT	MCC	BDT
FN035	20	**0.714**	**0.637**	0.577	0.508	-0.137	-0.129	**0.684**	**0.647**	0.078	0.080	0.866	0.955	0.890	0.947	0.134	**0.045**	0.110	0.053
FN096	24	**0.678**	**0.596**	0.546	0.473	-0.132	-0.123	**0.590**	**0.547**	0.038	0.054	0.957	0.948	0.948	0.941	**0.043**	0.052	0.052	0.059
FN339	24	**0.648**	**0.576**	0.546	0.473	-0.102	-0.103	**0.468**	**0.487**	0.038	0.054	0.941	0.926	0.885	0.934	0.059	**0.034**	0.115	0.105
FN242	23	**0.649**	**0.557**	0.545	0.475	-0.104	-0.082	**0.456**	**0.333**	0.008	0.044	0.975	0.851	0.975	0.851	**0.025**	0.074	**0.025**	0.066
FN315	21	**0.681**	**0.512**	0.582	0.473	-0.099	-0.039	**0.403**	**0.077**	0.068	0.054	0.831	0.450	0.763	0.450	0.169	0.149	0.237	0.149
FN104	22	**0.666**	**0.573**	0.555	0.484	-0.112	-0.089	**0.275**	**0.186**	-0.012	-0.016	0.912	0.778	0.808	0.778	0.088	0.141	0.192	0.222
FN110	22	**0.657**	**0.618**	0.588	0.508	-0.070	-0.111	**0.274**	**0.488**	0.117	0.102	0.684	0.895	0.500	0.895	0.316	0.384	0.500	0.550
FN094	23	**0.575**	0.473	0.571	**0.494**	-0.004	**0.021**	-0.013	-0.104	**0.113**	**0.112**	0.301	0.112	0.233	0.112	0.699	0.375	0.767	0.500
FN114	23	**0.576**	**0.478**	0.539	0.472	-0.037	-0.006	-0.076	-0.198	**-0.010**	**-0.002**	0.662	0.277	0.404	0.277	0.388	0.835	0.596	0.888
FN113	22	**0.575**	**0.516**	0.565	0.493	-0.010	-0.023	-0.109	-0.027	**0.067**	**0.056**	0.377	0.500	0.266	0.500	0.623	0.419	0.734	0.581
FN236	24	**0.535**	0.444	0.546	**0.473**	-0.011	**0.029**	-0.121	-0.265	**0.038**	**0.054**	0.463	0.152	0.320	0.152	0.537	0.610	0.680	0.723
FN452	22	**0.589**	**0.508**	0.560	0.488	-0.028	-0.020	-0.166	-0.135	**0.007**	**0.032**	0.639	0.419	0.448	0.419	0.361	0.755	0.552	0.848
FN017	23	0.471	0.430	**0.571**	**0.494**	**0.100**	**0.063**	-0.442	-0.285	**0.113**	**0.112**	**0.040**	**0.037**	**0.024**	**0.037**	0.960	0.960	0.976	0.963
FN425	23	0.429	0.345	**0.545**	**0.471**	**0.116**	**0.126**	-0.523	-0.599	**-0.011**	**0.011**	**0.049**	**0.012**	**0.049**	**0.012**	0.951	0.984	0.951	0.941
FN072	23	0.474	0.423	**0.571**	**0.494**	**0.097**	**0.071**	-0.528	-0.376	**0.113**	**0.112**	**0.024**	**0.059**	**0.019**	0.059	0.976	0.957	0.981	0.970
FN057	23	0.475	0.419	**0.571**	**0.494**	**0.096**	**0.075**	-0.540	-0.386	**0.113**	**0.112**	**0.022**	**0.030**	**0.012**	**0.030**	0.978	0.978	0.988	0.990
FN415	23	0.457	0.416	**0.571**	**0.494**	**0.114**	**0.078**	-0.556	-0.426	**0.113**	**0.112**	**0.022**	**0.022**	**0.012**	**0.010**	0.978	0.992	0.988	0.988

The analysis is based on common subsets of *all *CASP9 targets, with a minimum of 10 predictions in common. N, size of the common subset used in the comparison; MCC, Matthews Correlation Coefficient; BDT, the Binding Site Distance Test Score [36].P-value, the p-value for the paired Wilcoxon signed rank sum test using the raw scores; P-value (Z-score), the p-value for the paired Wilcoxon signed rank sum test using the Z-scores; 1 - p-value, 1 minus the p-value for the paired Wilcoxon signed rank sum test using the raw scores; 1-p-value (Z-score), 1 minus the p-value for the paired Wilcoxon signed rank sum test using the Z-scores. The table is sorted by the Mean MCC Z-score for groups with the best CASP9 groups at the top. The highest mean scores, mean Z-scores, significant p-values and significant 1-p-values are indicated in bold. Server groups are underlined.

**Table 10:** The mean MCC and BDT raw scores and Z-scores obtained by FunFOLD are compared with those obtained by the top CASP9 function prediction groups - as in Table 9 except for CASP9 targets containing *only metal ligands*, with a minimum of 6 predictions in common.

Group ID	N	Mean score for group		Mean score for FunFOLD		Difference in mean score		Mean Z-score for group		Mean Z-score for FunFOLD		P-value (raw score)		P-value (Z-score)		1 - p-value (raw score)		1 - p-value (Z-score)	
		
		MCC	BDT	MCC	BDT	MCC	BDT	MCC	BDT	MCC	BDT	MCC	BDT	MCC	BDT	MCC	BDT	MCC	BDT
FN114	6	**0.642**	**0.560**	0.408	0.343	-0.234	-0.217	**0.602**	**0.777**	-0.264	-0.275	0.947	0.970	0.911	0.947	0.053	**0.030**	0.089	0.053
FN094	6	**0.651**	**0.521**	0.530	0.428	-0.122	-0.092	**0.555**	**0.551**	0.210	0.159	0.860	0.860	0.791	0.860	0.140	0.140	0.209	0.140
FN452	6	**0.626**	**0.518**	0.492	0.397	-0.134	-0.121	**0.417**	**0.491**	-0.079	-0.104	0.911	0.860	0.911	0.911	0.089	0.140	0.089	0.089
FN242	7	**0.540**	**0.438**	0.450	0.367	-0.089	-0.071	**0.381**	**0.369**	-0.063	-0.004	0.911	0.736	0.911	0.799	0.089	0.265	0.089	0.201
FN339	7	**0.536**	**0.424**	0.450	0.367	-0.085	-0.057	**0.345**	**0.406**	-0.063	-0.004	0.853	0.735	0.799	0.896	0.147	0.265	0.201	0.104
FN096	7	**0.562**	**0.420**	0.450	0.367	-0.112	-0.053	**0.258**	**0.207**	-0.063	-0.004	0.853	0.663	0.735	0.853	0.147	0.337	0.265	0.147
FN236	7	**0.470**	**0.369**	0.450	0.367	-0.019	-0.002	**-0.009**	-0.065	-0.063	**-0.004**	0.606	0.606	0.606	0.500	0.394	0.394	0.394	0.500
FN057	6	0.371	0.250	**0.530**	**0.428**	0.158	0.178	-0.565	-0.770	**0.210**	**0.159**	0.109	**0.031**	0.156	**0.031**	0.891	0.969	0.844	0.969
FN072	6	0.360	0.239	**0.530**	**0.428**	0.170	0.189	-0.571	-0.817	**0.210**	**0.159**	0.109	**0.047**	0.156	**0.031**	0.891	0.953	0.844	0.969
FN425	7	0.281	0.226	**0.450**	**0.367**	0.169	0.142	-0.626	-0.546	**-0.063**	**-0.004**	0.209	0.089	0.109	0.140	0.791	0.911	0.791	0.860
FN017	6	0.233	0.201	**0.530**	**0.428**	0.297	0.227	-1.143	-1.008	**0.210**	**0.159**	**0.031**	**0.016**	**0.031**	**0.016**	0.969	0.984	0.969	0.984
FN415	6	0.197	0.172	**0.530**	**0.428**	0.333	0.256	-1.356	-1.214	**0.210**	**0.159**	** 0.031**	**0.016**	**0.031**	**0.016**	0.969	0.984	0.969	0.984

**Table 11:** The mean MCC and BDT raw scores and Z-scores obtained by FunFOLD are compared with those obtained by the top CASP9 function prediction groups - as in Table 9 except for CASP9 targets containing *non-metal ligands *or *metal and non-metal ligands *in the same binding site.

Group ID	N	Mean score for group		Mean score for FunFOLD		Difference in mean score		Mean Z-score for group		Mean Z-score for FunFOLD		P-value (raw score)		P-value (Z-score)		1 - p-value (raw score)		1 - p-value (Z-score)	
		
		MCC	BDT	MCC	BDT	MCC	BDT	MCC	BDT	MCC	BDT	MCC	BDT	MCC	BDT	MCC	BDT	MCC	BDT
FN035	17	**0.707**	**0.636**	0.582	0.516	-0.125	-0.019	**0.698**	**0.647**	0.056	0.105	0.888	0.951	0.922	0.940	0.112	**0.049**	0.078	0.060
FN096	17	**0.718**	**0.654**	0.582	0.516	-0.136	-0.138	**0.638**	**0.566**	0.056	0.105	0.934	0.827	0.920	0.858	0.066	0.073	0.080	0.412
FN315	17	**0.681**	**0.592**	0.582	0.516	-0.099	-0.076	**0.528**	**0.345**	0.056	0.105	0.785	0.741	0.798	0.694	0.215	0.259	0.202	0.306
FN242	16	**0.696**	**0.613**	0.582	0.522	-0.113	-0.091	**0.505**	**0.389**	0.014	0.093	0.934	0.795	0.947	0.795	0.066	0.205	0.053	0.205
FN339	17	**0.688**	**0.630**	0.582	0.516	-0.106	-0.114	**0.457**	**0.455**	0.056	0.105	0.836	0.847	0.741	0.824	0.164	0.153	0.259	0.176
FN110	17	**0.648**	**0.609**	0.582	0.516	-0.066	-0.093	**0.176**	**0.381**	0.056	0.105	0.710	0.878	0.518	0.741	0.290	0.122	0.482	0.259
FN104	17	**0.636**	**0.549**	0.582	0.516	-0.054	-0.033	**0.135**	0.006	0.056	**0.105**	0.644	0.463	0.573	0.391	0.356	0.537	0.427	0.609
FN236	17	0.563	0.481	**0.582**	**0.516**	0.019	0.035	-0.128	-0.262	**0.056**	**0.105**	0.377	0.134	0.295	0.122	0.623	0.866	0.705	0.878
FN094	17	0.551	0.461	**0.582**	**0.516**	0.031	0.055	-0.145	-0.263	**0.056**	**0.105**	0.122	**0.021**	0.183	**0.023**	0.878	0.979	0.817	0.977
FN017	17	0.551	0.500	**0.582**	**0.516**	0.031	0.016	-0.215	-0.103	**0.056**	**0.105**	0.290	0.259	0.202	0.215	0.710	0.741	0.798	0.785
FN452	16	**0.583**	0.519	0.582	**0.516**	-0.001	0.003	-0.253	-0.209	**0.014**	**0.093**	0.556	0.421	0.421	0.257	0.444	0.579	0.579	0.743
FN114	17	0.555	0.448	**0.582**	**0.516**	0.027	0.068	-0.263	-0.515	**0.056**	**0.105**	0.276	**0.046**	0.197	**0.030**	0.724	0.954	0.803	0.970
FN415	17	0.546	0.494	**0.582**	**0.516**	0.036	0.023	-0.271	-0.183	**0.056**	**0.105**	0.244	0.189	0.142	0.122	0.756	0.811	0.853	0.878
FN113	17	0.562	0.501	**0.582**	**0.516**	0.020	0.015	-0.333	-0.211	**0.056**	**0.105**	0.274	0.356	0.164	0.259	0.726	0.644	0.836	0.741
FN425	16	0.494	0.404	**0.582**	**0.516**	0.089	0.112	-0.434	-0.517	**-0.013**	**0.046**	0.147	**0.035**	0.132	**0.047**	0.853	0.965	0.868	0.953
FN057	17	0.507	0.468	**0.582**	**0.516**	0.075	0.048	0.569	0.323	**0.056**	**0.105**	0.060	0.148	**0.028**	0.080	0.940	0.164	0.972	0.836
FN072	17	0.506	0.473	**0.582**	**0.516**	0.076	0.043	0.592	0.328	**0.056**	**0.105**	0.094	0.183	**0.052**	0.095	0.906	0.274	0.948	0.726

In line with the analysis performed using the CASP8 dataset, we also tested the FunFOLD method using the CASP9 TS1 3D models obtained from the Zhang-Server, but again we saw no significant difference in performance by using a different starting model (results not shown). The results in Tables 12 and 13 compare the FunFOLD performance using CASP9 native structures with partial and extended binding site definitions respectively. In Table 12, no improvement is seen from using native structures, whilst Table 13 shows that a slight performance increase in mean scores can be achieved, however this is not significant.

**Table 12:** What is the effect on FunFOLD performance if the native structures are used for each target? A repeat comparison of FunFOLD performance versus the top 3 groups for at CASP9 using partial binding site definitions.

Group ID	N	Mean score for group		Mean score for FunFOLD		Difference in mean score		Mean Z-score for group		Mean Z-score for FunFOLD		P-value (raw score)		P-value (Z-score)		1 - p-value (raw score)		1 - p-value (Z-score)	
		
		MCC	BDT	MCC	BDT	MCC	BDT	MCC	BDT	MCC	BDT	MCC	BDT	MCC	BDT	MCC	BDT	MCC	BDT
FN035	22	**0.714**	**0.644**	0.621	0.541	-0.092	-0.103	**0.627**	**0.627**	0.387	0.197	0.649	0.869	0.784	0.889	0.351	0.131	0.216	0.111
FN096	25	**0.696**	**0.620**	0.617	0.534	-0.079	-0.086	**0.555**	**0.499**	0.404	0.250	0.674	0.850	0.594	0.771	0.326	0.150	0.406	0.229
FN339	25	**0.668**	**0.606**	0.617	0.534	-0.051	-0.072	**0.462**	**0.482**	0.404	0.250	0.614	0.837	0.500	0.771	0.386	0.163	0.500	0.229

**Table 13:** What is the effect on FunFOLD performance if the native structures are used for each target? A repeat comparison of FunFOLD performance versus the top 3 groups for at CASP9 using extended binding site definitions.

Group ID	N	Mean score for group		Mean score for FunFOLD		Difference in mean score		Mean Z-score for group		Mean Z-score for FunFOLD		P-value (raw score)		P-value (Z-score)		1 - p-value (raw score)		1 - p-value (Z-score)	
		
		MCC	BDT	MCC	BDT	MCC	BDT	MCC	BDT	MCC	BDT	MCC	BDT	MCC	BDT	MCC	BDT	MCC	BDT
FN035	21	**0.635**	**0.538**	0.585	0.520	-0.050	-0.018	**0.528**	**0.567**	0.465	0.462	0.684	0.620	0.636	0.695	0.316	0.380	0.364	0.305
FN096	24	**0.632**	**0.526**	0.585	0.516	-0.046	-0.010	0.468	0.343	**0.472**	**0.484**	0.678	0.605	0.450	0.406	0.322	0.395	0.550	0.594
FN339	24	**0.606**	0.501	0.585	**0.516**	-0.021	-0.015	0.426	0.302	**0.472**	**0.484**	0.614	0.439	0.386	0.332	0.386	0.561	0.614	0.668

### Examples of predictions

**Figure 8:**
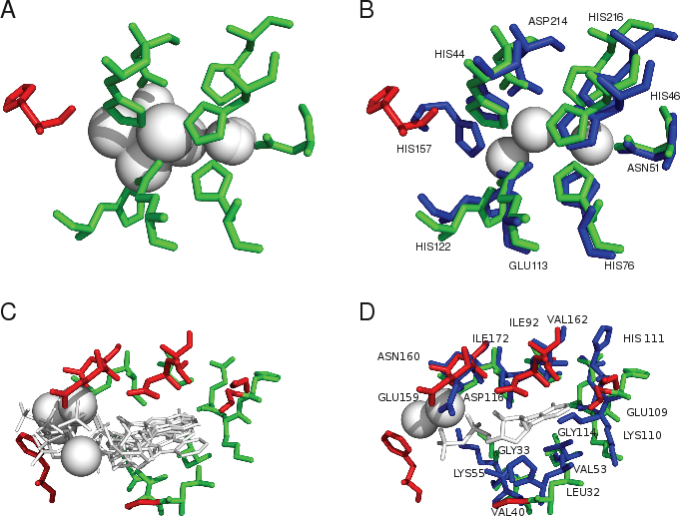
**Examples of binding site predictions from CASP8 targets using FunFOLD**. The green sticks represent residues in the model that FunFOLD has predicted as binding to the ligands. The red sticks represent residues that were not predicted or incorrectly predicted as potential ligand binding residues. The blue sticks represent the observed ligand binding site residues in the experimental structure. The white spheres and the thin white sticks represent ligands either predicted (A and C) or observed (B and D). A) An example of a good FunFOLD prediction for CASP8 target T0407 (3e38), with the predicted binding site residues and ligands shown. The FunFOLD method predicted both metals and SO4 in the ligand cluster (CL-3, ZN-6, SO_4_-2 and FE-2), with the centroid ligand predicted to be SO_4_. B): The predicted binding site for T0407 using the model superposed onto the experimental structure, with both the observed and predicted binding site residues shown. The observed binding site ligands are also shown (ZN - 3). The red sticks represent the under-prediction of HIS157. C) An example where FunFOLD under-predicts to a greater extent for CASP8 target T0483 (3dls). The predicted ligand binding site residues and ligands are shown. The FunFOLD method predicted the centroid ligand as a nucleotide (ANP), with the ligand cluster containing both nucleotides and metals (ANP-3, ATP-1, STU-1, ADP-1, MG-5 and MN-2). D) The predicted binding site of the top model for T0483 superposed onto the experimental structure, with both the observed and predicted binding site residues shown. The binding site ligands ADP and Mg-2 are also shown.

**Figure 9:**
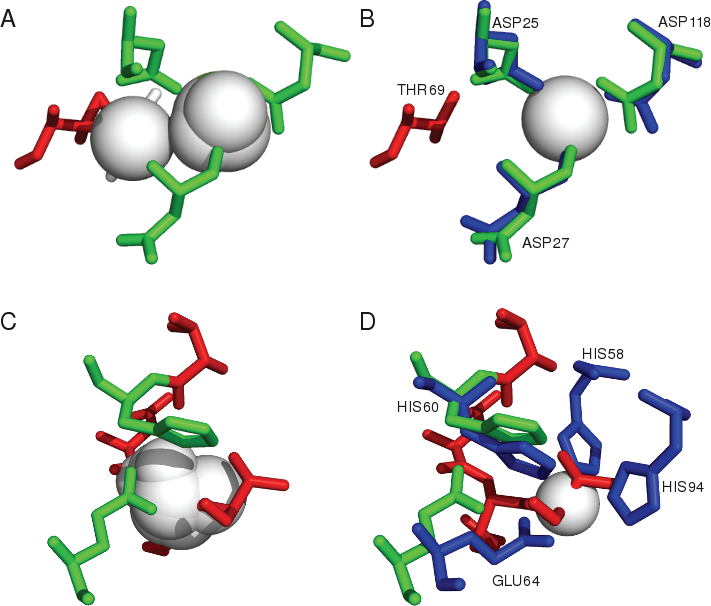
**Examples of binding site predictions from CASP9 targets using FunFOLD**. The green sticks represent residues in the model that FunFOLD has predicted as binding to the ligands. The red sticks represent residues that were not predicted or incorrectly predicted as potential ligand binding residues. The blue sticks represent the observed ligand binding site residues in the experimental structure. The white spheres represent ligands either predicted (A and C) or observed (B and D). A) An example of a good FunFOLD prediction for CASP9 target T0635 (3n1u). The predicted binding site residues and ligands are shown. The ligand cluster contains both metals and sulphates (CL-1, CA-2, SO_4_-2 and MG-4), with the centroid ligand predicted as MG. The red stick represents the over-prediction of residue THR69. B): The predicted binding site for T0635 using the model superposed onto the experimental structure. The observed and predicted binding site residues and the observed ligand (CA) are shown. The red sticks represent the over-prediction of THR69. C) An example where FunFOLD over-predicts to a greater extent for CASP9 target T0582 (3o14). The predicted binding site residues and ligands (ZN-1, CU-1, FE-3, FE2-1, MN-2) are shown. The centroid ligand in the ligand cluster is predicted to be ZN. D) The predicted binding site of the top model for T0582 superposed onto the experimental structure, with predicted and observer ligand binding site residues and the observed ligand (ZN) are shown.

If the FunFOLD method is provided with a good quality model and a list of templates that are structurally similar containing biologically relevant ligands, then highly accurate predictions can be achieved (Figure 8A and 8B; Figure 9 A and 9B).

Figure 8A and 8B represents accurate predictions for the CASP8 target T0407 (PDBID 3e38) with an MCC score of 0.941 and BDT score of 0.888. For comparison, the prediction by group FN202 was also very accurate with an MCC = 0.902 and BDT = 0.827, however the MCC and BDT scores for group FN407 were only 0.579 and 0.563, respectively. Group FN293 did not make a prediction for target T0407.

Analysing the prediction for T0407 in more detail, the FunFOLD method correctly predicted the binding site as being a metal binding site and the observed zinc ligands to be in the binding pocket. However, the method also under predicted one residue - HIS157. This under prediction occurred because the aromatic ring of the histidine residue was orientated away from the binding site in the model, and therefore HIS157 received a low vote of 1/13 (7.69%).

Figure 8C and 8D illustrate where FunFOLD did not succeed in predicting all of the correct residues within the binding site of CASP8 target T0483 producing many false negatives, as well as incorrectly predicting other residues which are not officially considered to be involved in binding to the ligand (false positives). Despite these errors the FunFOLD prediction for T0483 achieved an MCC = 0.616 and a BDT = 0.503, which were higher scores than those produced by FN202 (MCC = 0.453; BDT = 0.492). However, group FN293 achieved an MCC = 0.898 and a BDT = 0.876, and group FN407 achieved an MCC = 0.904 and a BDT = 0.887, for the same target. The FunFOLD method also over predicted 3 residues in target T0483 as ligand binding residues: 35, 37 and 112. The votes for residues 35 and 112 were just above the threshold of 25% at 30.77%, whilst residue 37 had 61.54% of the votes.

According to the ModFOLDclust2 [28] predictions, the global model quality score for the model used in the prediction for target T0483 is ~0.6 (scores >0.4 indicate the fold has a good probability of being correct). However, the local model quality around the binding site is comparatively bad. Thus, FunFOLD did not predict the observed residues 33, 110, 111, 114, 116, 159 and 162. Residues 33, 110 and 159 simply did not receive enough ligand votes to be considered; with residue 33 falling below the cut-off with a vote of 15.39%, 110 getting a low vote of 7.69% and 159 getting a vote just below the cut-off of 23.08%. In addition FunFOLD did not identify any ligand contacts with residues 116 and 162, and residues 111 and 114 were identified in an alternative binding site cluster.

Figure 9A and 9B also represents accurate predictions for CASP9 target T0635 (PDB ID 3n1u), with an MCC score of 0.864 and a BDT score of 0.759. For comparison the Zhang group (FN096) and the I-TASSER-FUNCTION method (FN339) achieved and MCC = 0.603 and a BDT = 0.392, firestar (FN315) achieved an MCC = 0.770 and BDT = 0.617, whilst the Jones-UCL group (FN104), the Sternberg group (FN110), the LEE group (FN114) and the gws server (LEE server FN236) all achieved an MCC = 1.0 and BDT = 1.0.

When a more detailed analysis of the predictions for T0635 was carried out, the FunFOLD method correctly predicted the binding site as being a metal binding site, the correct location of the binding site and the three binding site residues. However, the method over-predicted one residue THR69. This over prediction occurred because the residue was in contact with three ligands (SO_4_-2 and CL-1), which were not well superposed onto the ligand cluster receiving a vote of 3/9 (33.33%).

Figure 9C and 9D, showing CASP9 target T0582 (PDB ID 3o14), again represents a case where FunFOLD did not succeed in predicting all of the binding site residues and incorrectly predicted other residues that were not part of the official binding site. Despite these errors FunFOLD achieved an MCC = 0.395 and BDT = 0.348. The FunFOLD method correctly predicted residues 60 and 64, but over-predicted residues 59, 115, 116, and 117 and failed to predict binding site residues 58 and 94. Residue 59 received 2/8 votes, residues 115 and 116 received 8/8 votes and residue 117 received 7/8 votes. The under-predicted residues 58 received 1/9 votes, while residue 94 was not predicted to be in contact with the ligand cluster.

According to ModFOLDclust2 [28] predictions, the global model quality score for the model used in the prediction for CASP9 target T0582 = 0.466, which is a reasonable global model quality score. However yet again, the local model quality around the binding site for this model is comparatively bad. This resulted in both over and under-predictions, with residues 115, 116 and 117 being poorly modelled.

## Discussion

In this study we describe a novel method, FunFOLD, for the prediction of ligand binding site residues, which shows a significant improvement over all of the true server methods that were tested at CASP8, as well as the predictions from one of the top manual groups - FN202. In addition, the method was tested on the CASP9 set and was found to be competitive with the top server groups and statistically inseparable in performance from most of the top manual groups. Whilst a prototype version of a server was tested in the CASP9 function prediction category (IntFOLD-FN - FN425), we have since improved the reliability of the FunFOLD server and the current automated implementation more closely resembles the performance of our manual function predictions (McGuffin - FN094).

The performance of all methods was measured using the standard Matthews Correlation Coefficient (MCC) [35], on both the CASP8 and CASP9 function (FN) targets. The mean MCC Z-scores for FunFOLD, were shown to be higher than the mean MCC Z-scores for all other methods tested at CASP8 (Table 1). The FunFOLD predictions were an improvement upon those made by the Lee manual group (+1% MCC), the Lee server group (+4%) and the Sternberg group (+13%), which were the top groups tested during CASP8. The improvement over the Sternberg group on the CASP8 data set is statistically significant at the 99% level according to the Wilcoxon Signed rank sum test. The FunFOLD method is also shown to be competitive with all methods tested at CASP9 and shows no statistically significant difference with the top ranking server methods: I-TASSER-FUNCTION and firestar [4] (Tables 4, 5, 6 and 7). However, a statistically significant improvement was seen over the 3DLigandSite methods that were tested at CASP9, at the 99% level according to the Wilcoxon Signed rank sum test.

Intuitively, the quality of the starting 3D model that is used for the prediction of the ligand binding site will have an effect on the accuracy of results. Thus, in this paper for the CASP8 analysis we used ModFOLDclust2 model quality assessment method [28] to select high quality input models. However, we also tested FunFOLD using alternative 3D models that were submitted by the top function prediction groups and found that the improvement in MCC scores was maintained - the Lee manual method was improved upon by +2%, Lee server by +3% and the Sternberg group by +10%, which was again shown to be statistically significant. In addition, when native structures were used, as might be expected, the improvement in performance was maintained.

On the CASP9 data set we tested starting models from both our IntFOLD-TS server and from the better performing Zhang-Server, however again no significant difference in performance was seen. Furthermore, whilst using the native structures improved performance marginally in some cases, in the majority of cases the increases were not significant. According to these benchmarks, the results indicate that selecting an alternative starting model does not have much of an influence. Therefore, where there has been a significant improvement over other groups, this must have arisen from the FunFOLD algorithm itself, rather than from improved initial model selection.

One of the top ligand binding site prediction servers tested at CASP9 was the I-TASSER-FUNCTION server method (Zhang group), which also relies on 3D model-to-template superposition, for binding site residue prediction. However, in addition to global model to template superposition, the I-TASSER-FUNCTION method also carries out local alignment of the proposed binding site region, in order to improve local superposition. In light of the results shown in Figures 8 and 9, local superposition scores, such as those used in I-TASSER-FUNCTION, could be adopted which may help to improve future versions of FunFOLD. However, in our benchmarking on the CASP9 set we could measure no significant increase in performance of the I-TASSER-FUNCTION method over FunFOLD.

At the time of writing the I-TASSER-FUNCTION server is currently not publicly available. Therefore, arguably the top ranking publicly available server tested at CASP9 was firestar [4], which predicts residue conservation in target sequences based on PSI-BLAST alignments to the large catalogue of sites in PDB structures contained in the FireDB [30]. However, the firestar method is not currently available as a standalone program and again we could measure no significant performance gain over the FunFOLD method.

The FunFOLD method is clearly also competitive with the top manual groups that were tested in CASP8, however no manual intervention is required for our approach. Furthermore, the method significantly outperforms each of the true server methods tested at CASP8. Whilst the Lee server group (FN293) was mostly automated, the group was counted in the extended deadline category and the authors reported a small amount of human intervention [3], hence in this study we have considered group FN293 in CASP8 as a non-server group.

The FunFOLD method also significantly outperformed one of the top manual groups (FN202) and since CASP8, the authors have developed a fully automated publicly available server, called 3DLigandSite [15], variations of which participated in CASP9. The authors reported that the predictions from the 3DLigandSite server were comparable in performance to their manual predictions at CASP8 [15], therefore in this study we can consider predictions from group FN202 to be the gold standard for fully automated ligand binding residue prediction for testing on the CASP8 data set.

The standalone FunFOLD software uses similar input data to the version of the FINDSITE [11] software that is currently available to download. For both programs, a 3D model and a list of templates is required, however, the methods differ in the output produced; the FunFOLD method outputs binding residue predictions in CASP FN format, where as the FINDSITE method outputs a list of putative locations for the centre of each binding pocket i.e. locations in 3D space rather than binding site residues. Thus, the FunFOLD software cannot be directly compared to the FINDSITE software as they both produce different output. Furthermore, the FINDSITE dataset [11] cannot be directly used in our analysis, as the location of the binding site residues for each template is not defined and would have to be predicted, adding potential for errors in methods comparison. However, the latest FINDSITE-DBDT method did compete in CASP9, but to our knowledge the server is not publicly available. The current implementation of FunFOLD, the prototype version of the server (FN425) and our manual prediction group (FN094) performed statistically significantly better than the FINDSITE-DBDT method on the CASP9 data set [27].

The FunFOLD method uses a similar procedure to that carried out by the most successful prediction groups participating CASP; the 3D input model is superposed onto structurally similar ligand containing PDB files and the putative binding residues are then determined. However, the FunFOLD method uses a novel, fully automated approach for both identifying clusters of ligands and determining putative binding site residues. The novel ligand residue voting method used in FunFOLD reduces the rate of over predictions, which appears to be one of the main problems with many structure based approaches.

There are several caveats to consider when benchmarking methods for the prediction of protein ligand binding residues. Firstly, uncertainties can arise if there are several ligand binding sites within a protein either predicted or observed. For this analysis we only considered the binding residues that were defined by the CASP assessors and for each target only one binding site was defined. Secondly, the inherent flexibility of proteins may make it difficult to determine which residues are actually in contact with the ligand. This is further exacerbated if the binding site is located in a disordered region of a protein. Thirdly, the definition of the distance cut-off for a residue that is in contact with a ligand is the Van der Waals radii + 0.5 Å, but this definition is subjective. Finally, there may be ambiguity about whether the ligand bound to the solved structure is the protein's ideal ligand. Thus a ligand used in a prediction may not necessarily be incorrect. Indeed one binding site may bind more than one ligand.

Each of these issues creates difficulties for the fair assessment of methods as defining a list of observed binding site residues may be subjective. Some of these issues were addressed by the exclusion of "neutral residues" in the CASP 8 analysis [21], which have also been excluded in this analysis for the CASP8 data (the CASP8 assessors defined neutral residues as those which would potentially bind to an alternative ligand, but which were not observed binding to the alternative ligand within the solved 3D structure). In CASP9 the assessors used two classifications for binding sites - partial and extended. From the official analysis there was not a significant difference in assessment, if methods were analyzed using either partial or extended binding site definitions. However, the use of the MCC statistic for assessment does compound some of these issues and the prediction of a binding residue that is defined as incorrect, but which is nevertheless close to the observed binding pocket will therefore obtain the same score as a random incorrect prediction.

Therefore we recently proposed a novel scoring method - the Binding-site Distance Test (BDT) score, which addresses some of the shortcomings of using MCC scores, whilst maintaining the advantages [36]. Predicted residues that are close to the observed residues will obtain a higher BDT score than more distant predictions. The BDT score was used by the CASP9 assessors, in addition to the MCC score, to investigate if it caused a significant difference in the rankings of the methods but no significant changes in the grouping of top methods were reported [27]. In this analysis, the list of top groups identified using BDT scoring is again roughly in agreement with the top groups obtained using the MCC scoring; however the ranking of some of the less accurate methods does appear to change. Using the mean BDT score, we also see higher scores for FunFOLD compared with all methods tested on CASP8 on equivalent subsets of data and the difference is again significant for all but the top two manual groups (Figure 2, Figure 6 and Table 1). When the CASP9 predictions are analysed using the mean BDT scores the FunFOLD method is ranked below the top two server methods I-TASSER-FUNCTION and firestar [4], however again the difference in performance is not significant.

An obvious way of improving future versions of the FunFOLD software would be to optimize the voting threshold for the inclusion of predicted residues. Furthermore, predicting the ligand binding site residues on multiple models and then pooling the results may help to increase accuracy. The specific physiochemical properties of the residues could be studied, such as charge and polarity, and residues exhibiting more favorable physiochemical properties for binding to a ligand could be weighted more heavily in the residue voting process. A prediction of the enzymes functional family could be performed, with the prediction then used to weight residues that bind to ligands that occur more often within these families, more heavily in the voting process. In addition to undertaking global model-to-template superpositions, local superposition of the binding site regions could also be carried out to increase accuracy. As previously mentioned this was carried out by one of the top server groups at CASP9 (I-TASSER-FUNCTION - FN339).

A general function prediction quality assessment tool could also be developed in order to weight predictions or provide probabilities scores for individual residues. Features of the quality assessment might include: the type of ligands within the cluster, with clusters containing a large number of similar ligands receiving a higher score; the distance of the superposed ligands from the centroid ligand within the cluster, with clusters containing ligands that are superposed perfectly receiving higher scores; the global and local model quality scores of the starting 3D model could also be factored into the analysis using our ModFOLD methods [28,37], with residues in poorly modeled regions down weighted; the probability of bound residues occurring in disordered regions could also be considered by integrating our DISOclust results [38], with residues in regions of high disorder receiving an appropriate weighting. In future, each of these features could be integrated into an automated quality assessment tool in order to produce more appropriate confidence scores which could be used for ranking binding residue predictions.

## Conclusion

The FunFOLD software implements a competitive method for the prediction of protein binding site residues, which can also be used to determine the putative ligands interacting with a protein. The method is available as a standalone program, which can be used to predict binding residues and ligands based on user supplied 3D models and template lists. We also provide access to FunFOLD via a simple web server, which only requires users to supply an amino acid sequence.

## Availability and requirements

• **Project name**: FunFOLD

• **Project homepages**: The standalone software can be downloaded from http://www.reading.ac.uk/bioinf/downloads/. The web server can be accessed at http://www.reading.ac.uk/bioinf/FunFOLD/. The FunFOLD method is also part of the IntFOLD server pipeline: http://www.reading.ac.uk/bioinf/IntFOLD/

• **Operating system(s)**: Platform independent (Linux preferred)

• **Other requirements**: The standalone version requires a recent version of Java, a recent version of PyMOL (http://www.pymol.org), and the TMalign program (http://zhanglab.ccmb.med.umich.edu/TM-align).

• **Licence**: freely available

## Conflict of interest

The authors declare that they have no competing interests.

## Authors' contributions

DBR contributed code, carried out testing of the system, carried out benchmarking, contributed figures and drafted the manuscript. SJT carried out testing of the system, and contributed text and figures to the manuscript. LJM conceived the idea, produced the executable, carried out statistical data analysis, contributed text and figures to the manuscript and carried out final editing of the manuscript. All authors read and approved the final manuscript.
